# α-Mangostin Competing the Menaquinone-Binding Sites of NDH-2 to Block the Electron Transfer at the Quinone Pool of *Staphylococcus aureus*

**DOI:** 10.3390/antibiotics15050509

**Published:** 2026-05-18

**Authors:** Meifang Zhang, Jianing Hu, Yu Wang, Liaolongyan Luo, Ganjun Yuan

**Affiliations:** 1 Biotechnological Engineering Center for Pharmaceutical Research and Development, Jiangxi Agricultural University, Nanchang 330045, China; 0202023095@stu.jxau.edu.cn (M.Z.);; 2 Laboratory of Natural Medicine and Microbiological Drug, College of Bioscience and Bioengineering, Jiangxi Agricultural University, Nanchang 330045, China

**Keywords:** *α*-mangostin, NDH-2, respiratory chain, menaquinone, mechanism, flavonoid

## Abstract

**Background/Objectives**: *α*-Mangostin, a natural product from *Garcinia mangostana* L, presents very strong antibacterial activity in plant flavonoids against *Staphylococcus aureus*. Recently, it was reported that the quinone pool is a key target of *α*-mangostin against Gram-positive bacteria. Here, the detail centering this action mechanism of *α*-mangostin killing *S. aureus* was further explored. **Methods**: The interactions between α-mangostin and type II NADH:quinone oxidoreductase (NDH-2), a key enzyme in the respiratory chain, were explored through the enzyme kinetic experiments, fluorescence analyses, and molecular simulation. Simultaneously, the effect of α-mangostin on membrane potential was also investigated as a possible non-enzymatic mechanism. **Results**: it was found that *α*-mangostin mainly competes the menaquinone-binding sites of NDH-2 with menaquinone, and the half-maximal inhibitory concentration (IC_50_) of *α*-mangostin on NDH-2 is 4.95 μM. Fluorescence analyses indicated that *α*-mangostin can spontaneously bind to NDH-2 to form an *α*-mangostin–NDH-2 complex. Subsequently, molecular simulation further showed that *α*-mangostin can dock to the menaquinone-binding sites of NDH-2. In addition, non-enzymatic mechanism showed that *α*-mangostin can cause membrane potential depolarization and disrupt the proton motive force balance, thereby promoting the cell-membrane destruction of *S. aureus*. **Conclusions**: *α*-Mangostin can mainly interact with the amino acid residues at the menaquinone-binding pocket of NDH-2 to block the electron transfer at the quinone pool in the respiratory chain of *S. aureus*, which will hinder the energy supply and act synergistically with cell membrane damage, ultimately leading to the death of *S. aureus*. Simultaneously, it once again proves that the quinone pool is a key target of plant flavonoids against Gram-positive bacteria.

## 1. Introduction

The emergence of multidrug-resistant (MDR) bacteria has brought severe challenges to global health. Methicillin-resistant *Staphylococcus aureus* (MRSA) is one of the main MDR bacteria causing mortality, and it is the predominant source of bloodstream, skin, and soft tissue infections [1,2]. Although some antibiotics have been approved for the treatment of MRSA infection, the discovery of new drugs—especially from natural products with unique antimicrobial mechanisms—remains essential for treating infections caused by MRSA [3].

**Figure 1:**
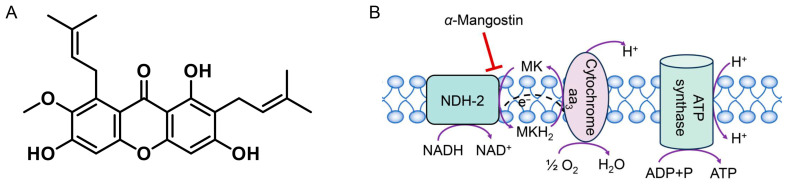
Structure of α-mangostin (**A**) and diagram of α-mangostin acting on NDH-2 in the respiratory chain of *S. aureus* (**B**).

Plant flavonoids, widely distributed in various plants and demonstrated by favorable safety profiles, have been drawing increasing attention to their antibacterial potential. Some of them not only show stronger antibacterial activity than clinical antibiotics, but also present synergistic effects in combination with antimicrobial agents and even reverse antibiotic resistance [4,5,6]. *α*-Mangostin (Figure 1A) is a flavonoid, belonging to the subclass of xanthones, from *Garcinia mangostana* L [7]. It presents very strong antibacterial activity in plant flavonoids against *Staphylococcus aureus*, and has been studied as an antibacterial agent against MRSA since the 1980s [8,9]. It is indicated the cell membrane is an important target of *α*-mangostin against MRSA [10], but its effects on the respiratory chain were not involved. Recently, a piece of research suggests that a key target for plant flavonoids inhibiting Gram-positive bacteria is the quinone pool of the respiratory chain, which likely involves multiple mechanisms including some enzyme and non-enzyme mechanisms [11].

The quinone pool is a key hub of the electron transfer on the respiratory chain of *S. aureus*. In front of the quinone pool, NDH-2 is a key enzyme that mediates electron transfer from NADH to menaquinone [12,13]. Namely, it can catalyze the reduction of menaquinone, which is the rate-limiting step of electron transfer in the respiratory chain. The interaction between NDH-2 and the quinone pool helps sustain the NADH/NAD^+^ redox balance, ultimately driving ATP synthesis and ensuring the energy supply of *S. aureus* [14,15]. As NDH-2 is the only respiratory NADH dehydrogenase in *S. aureus*, the inhibition to NDH-2 can eventually lead to bacterial cell death [16]. Simultaneously, preliminary molecular docking shows that *α*-mangostin can bind to the active pocket of NDH-2. Therefore, the present research investigated the interactions between *α*-mangostin and NDH-2 of *S. aureus*, together with potential non-enzymatic mechanisms, to elucidate the details by which this highly potent antibacterial flavonoid targets the quinone pool of Gram-positive bacteria.

## 2. Results

### 2.1. Minimum Inhibitory Concentrations (MICs) and Minimum Bactericidal Concentrations (MBCs)

The MICs of *α*-mangostin against *S. aureus* ATCC 25923 and MRSA ATCC 33592 was determined using the microbroth dilution method. The MICs of *α*-mangostin against *S. aureus* ATCC 25923 and MRSA ATCC 33592 are 2 μg/mL and 0.5 μg/mL, respectively. Based on the MIC results, their MBCs were further measured. The MBCs of *α*-mangostin against *S. aureus* ATCC 25923 and MRSA ATCC 33592 are 64 μg/mL and 4 μg/mL, respectively. These results suggest that *α*-mangostin has strong antibacterial activity against *S. aureus*. Moreover, the determination of MIC can better design drug concentrations.

### 2.2. Protein Purification and Enzymatic Characterization

**Figure 2:**
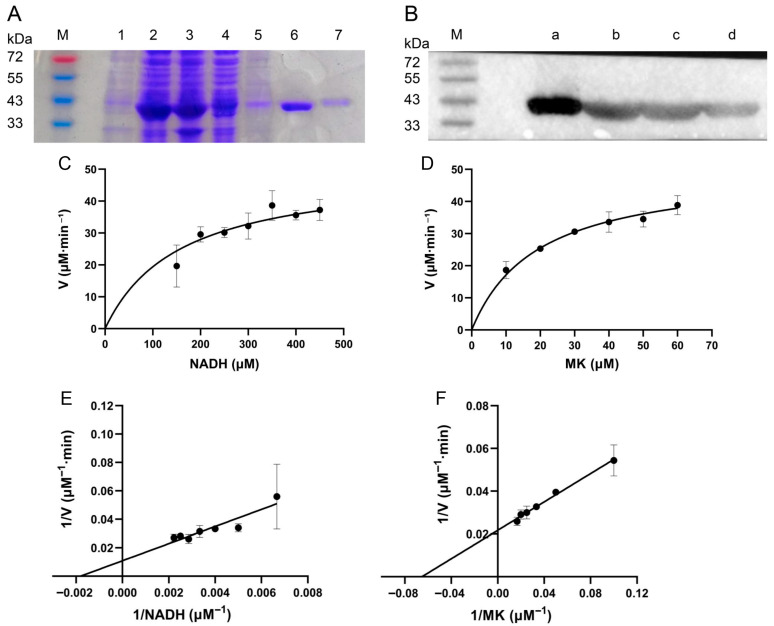
Heterologous expression and enzymatic characterization of NDH-2 (*n* = 3). (**A**) SDS-PAGE of purified NDH-2: 1: uninduced without IPTG; 2: supernatant; 3: precipitate; 4: flow-through; 5: 20 mM imidazole; 6: 75 mM imidazole; 7: 100 mM imidazole. (**B**) Western blot of NDH-2: a: Purified protein; b~d: supernatant. (**C**,**D**) Michaelis–Menten plots of the enzyme against different substrate concentrations. (**E**,**F**) Lineweaver–Burk plots of NDH-2.

The genome of *S. aureus* encodes two NDH-2 isozymes, namely NDH-2A and NDH-2B. NDH-2A has an irreplaceable function, its inhibition directly blocks the oxidation of NADH in the respiratory chain, leading to the collapse of bacterial energy metabolism [14]. However, the effect of NDH-2B inhibitors is easily evaded by bacteria. Therefore, NDH-2A was chosen for cloning. To investigate the effect of *α*-mangostin on NDH-2, we successfully achieved in vitro expression of NDH-2 through transformation in *E. coli* BL21(DE3) followed by IPTG induction. SDS-PAGE analysis (Figure 2A) revealed a distinct protein band at approximately 43 kDa. Furthermore, Western blot analysis (Figure 2B) confirmed the expression of the target protein. Due to the protein carrying a 6 × His tag, Ni^2+^-NTA agarose affinity chromatography combined with concentrator ultrafiltration was employed to obtain a highly purified protein sample that suitable for subsequent experiments. UV spectrophotometric analysis of the protein sample showed no absorption peak at 450 nm, while the purified enzyme solution was yellow, indicating that the heterologously expressed NDH-2 contains sub-stoichiometric amounts of FAD, which an important cofactor in the NDH-2 protein structure. Fortunately, after reconstitution by adding natural FAD in vitro, the measured kinetic parameters of NDH-2 showed no significant difference from the reported kinetic parameters of natural NDH-2 from *S. aureus*, and its substrate binding properties and catalytic activity were consistent with those of NDH-2 in the natural state. It truly reflects that the addition of FAD to the reaction system allowed the enzymatic reaction to proceed normally; the effectiveness of this refactoring method has been confirmed by existing research conclusions [16].

The natural oxidant for NDH-2 in the membrane of *S. aureus* is menaquinone [13]. Therefore, in this experiment, menaquinone was used as the oxidant to evaluate the NADH:menaquinone oxidoreductase activity of purified NDH-2 in the presence of NADH, and several enzymatic kinetic characteristics were determined (Figure 2C–F). Under the conditions of 30 °C and pH 7.5, the NADH:menaquinone oxidoreductase activity dependent on either NADH or MK followed simple Michaelis–Menten kinetics. The K_m_ value for NADH was 158 ± 40 μM, and for MK it was 19.3 ± 1.9 μM, which is consistent with previously reported values [17]. The successful determination of these enzymatic kinetic parameters indicates that NDH-2 effectively oxidizes NADH to NAD^+^. Thus, the purified NDH-2 can be utilized for investigating the antibacterial mechanism of α-mangostin.

### 2.3. Inhibitory Effect of α-Mangostin on NDH-2 Activity

**Figure 3:**
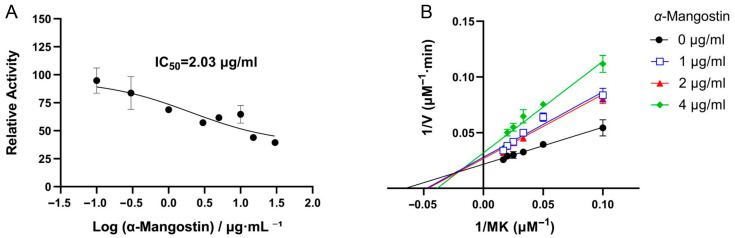
Inhibitory effect of *α*-mangostin on NDH-2. (**A**) Effect of *α*-mangostin on NDH-2 activity in the presence of 40 μM MK. (**B**) Kinetic analysis of NDH-2 inhibition by *α*-mangostin (Lineweaver–Burk plot). The experiment was performed in three biological replicates, and the data are presented as mean ± SD (*n* = 3).

The relationship between the relative activity of NDH-2 and different concentrations of *α*-mangostin is shown in Figure 3A. Data analysis revealed that the half maximal inhibitory concentration (IC_50_) of *α*-mangostin for NDH-2 is 2.03 ± 0.09 μg/mL (4.95 μM). HQNO, a known effective inhibitor of NDH-2, has a reported IC_50_ of 7.3 ± 1.2 μM [18]. Therefore, *α*-mangostin can be considered to exhibit stronger inhibitory efficacy against NDH-2 in vitro compared to the positive control, suggesting its potential as a promising drug candidate which targets NDH-2.

To further elucidate the inhibitory mechanism of *α*-mangostin on NDH-2, enzyme kinetic inhibition assays were performed (Figure 3B). The fitted curves for different concentrations of *α*-mangostin intersect in the second quadrant, and with increasing inhibitor concentration, the K_m_ value gradually increases while the V_max_ value decreases. These results indicate that *α*-mangostin acts as a mixed-type inhibitor of NDH-2. The inhibitor constant (K_i_), also known as the dissociation constant of the enzyme inhibitor complex, reflects the binding affinity between the enzyme and the inhibitor. A lower K_i_ value indicates stronger inhibition. Experimental results show that the competitive inhibition constant (K_ic_) of *α*-mangostin for NDH-2 is 1.73 μM, and the noncompetitive inhibition constant (K_in_) is 5.70 μM. The significantly lower K_ic_ value compared to K_in_ suggests that the competitive inhibition mode predominates in the mixed-type inhibition, indicating that *α*-mangostin can interact with both free NDH-2 and the NDH-2–MK complex, but exhibits stronger binding to free NDH-2. Although the kinetic characteristics conform to mixed inhibition, α-mangostin mainly exhibits competitive dominant mixed inhibition against NDH-2. Subsequent fluorescence spectroscopy and molecular docking both confirm that α-mangostin binds to NDH-2 rather than the NDH-2–MK complex, suggesting that α-mangostin competitively binds to the menaquinone site of NDH-2.

### 2.4. Analysis of the Binding Properties Between α-Mangostin and NDH-2

**Figure 4:**
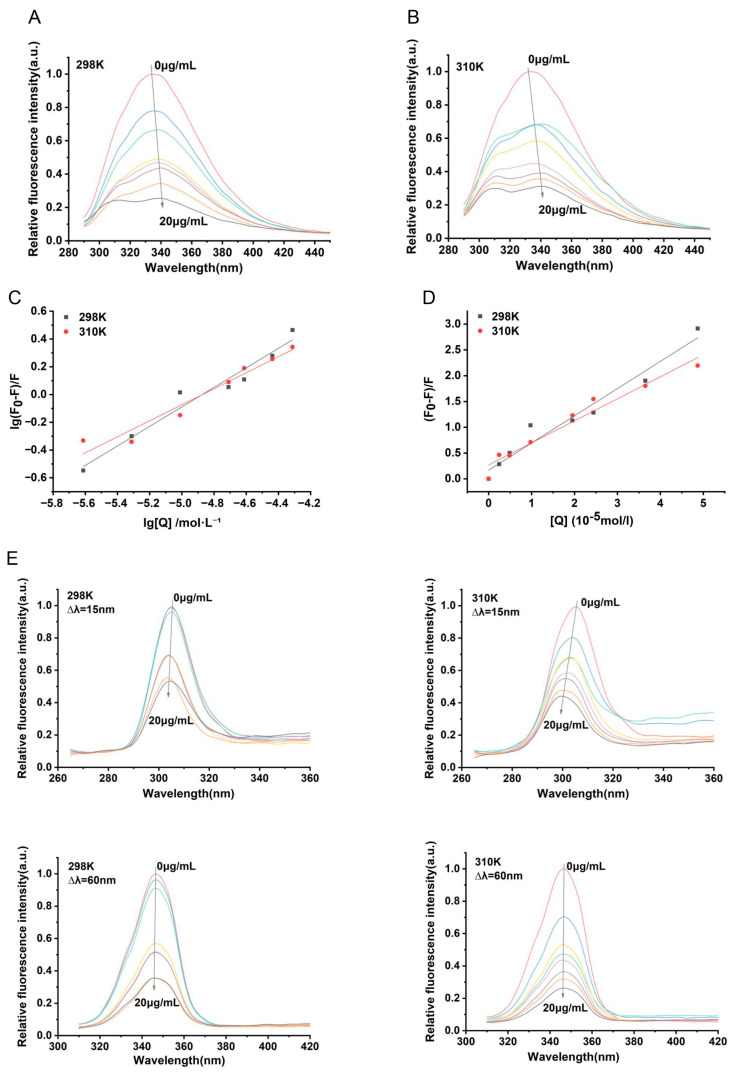
Fluorescence analysis diagram of *α*-mangostin quenching NDH-2. (**A**,**B**) Fluorescence emission spectra of NDH-2 quenched by *α*-mangostin at different temperatures. (**C**) Double logarithmic plots of *α*-mangostin quenching NDH-2 at 298 K and 310 K. (**D**) Stern–Volmer plots of *α*-mangostin quenching NDH-2 at 298 K and 310 K. (Due to slight fluctuations in the fluorescence signal, the data show minor dispersion, but generally exhibit an obvious concentration-dependent pattern, consistent with the characteristic law of static quenching. (**E**) Synchronous fluorescence spectra of *α*-mangostin quenching NDH-2 Tyr and Trp at 298 K and 310 K respectively (Δ*λ* = 15 nm for Tyr, Δ*λ* = 60 nm for Trp).

The intrinsic fluorescence properties of proteins, attributed to amino acids such as tryptophan (Trp) and tyrosine (Tyr), are highly sensitive to changes in their microenvironments. Therefore, fluorescence spectroscopy can be employed to analyze the binding characteristics and interactions between proteins and drug molecules [19]. With the excitation wavelength fixed at 280 nm, the fluorescence emission spectrum of NDH-2 was measured. As shown in Figure 4A,B, the maximum fluorescence intensity peak of NDH-2 appeared near 335 nm. Under incubation conditions at 298 K and 310 K, the fluorescence intensity of NDH-2 gradually decreased with increasing concentrations of *α*-mangostin, indicating that *α*-mangostin interacts with Trp and Tyr residues in NDH-2 in a manner that quenches their fluorescence.

To further investigate the quenching mechanism of NDH-2 by *α*-mangostin, we calculated the quenching constant (*K*_sv_), bimolecular quenching rate constant (*K*_q_), binding constant (*K*_a_), number of binding sites (*n*), enthalpy change (ΔH), entropy change (ΔS), and Gibbs free energy (ΔG) based on fluorescence spectra obtained at 298 K and 310 K. The results are summarized in Table 1, and the linear fitting curves are shown in Figure 4C,D. As seen in Table 1, the *K*_sv_ values for the quenching of NDH-2 by *α*-mangostin decrease with increasing temperature. Additionally, the *K*_q_ values exceed the maximum dynamic quenching constant (2.0 × 10^10^ L/mol/s), indicating that the quenching mechanism is static quenching. During this process, a *α*-mangostin–NDH-2 complex is formed, and the stability of this complex decreases with rising temperature, suggesting that the binding affinity between *α*-mangostin and NDH-2 weakens as temperature increases. The values of *n* at both 298 K and 310 K were greater than 0.5, indicating a single binding site for *α*-mangostin on NDH-2. It reflects the average properties of fluorescence measurements. Importantly, this observation is not inconsistent with the molecular docking results, which indicate the presence of a single specific binding site in the menaquinone binding pocket. This suggests the presence of a specific binding site for *α*-mangostin in the NDH-2, which may be located within the enzyme’s active center. Furthermore, the *K*_a_ for the interaction between *α*-mangostin and NDH-2 are all below 10^4^ L·mol^−1^, indicating weak binding. As temperature increases, the *K*_a_ values decrease, which aligns with the theoretical expectation that higher temperatures reduce the stability of protein–quencher complexes and lead to lower binding constants in static quenching. This result further confirms that the binding affinity between *α*-mangostin and NDH-2 diminishes with increasing temperature. In addition, in the binding process between *α*-mangostin and NDH-2, the values of ΔG, ΔH, and ΔS are all negative, indicating that the binding is spontaneous. Hydrogen bonds and van der Waals forces play dominant roles in the interaction. Moreover, the negative ΔH value signifies an exothermic reaction, which contributes to the weakening of binding and the observed decrease in *K*_a_. This finding is consistent with the conclusions drawn above.

To investigate the microenvironmental information of Tyr or Trp residues after the binding of *α*-mangostin to NDH-2, synchronous fluorescence spectra at different *α*-mangostin concentrations were measured, with Δλ between emission and excitation wavelengths set at 15 nm and 60 nm, respectively. The results (Figure 4E) show that as the concentration of *α*-mangostin increases, all maximum emission wavelengths (λ_max_) exhibit a blue shift. This indicates that *α*-mangostin specifically binds to NDH-2, leading to a decrease in the polarity and freedom of the microenvironment around the Trp and Tyr residues of the protein. It is thus inferred that the binding site is located in the hydrophobic region of the protein, where *α*-mangostin interacts stably with NDH-2 through hydrophobic interactions and hydrogen bonding, which is subsequently supported by molecular docking experiments. At 298 K, the blue shifts in λ_max_ for both Δλ = 15 nm and Δλ = 60 nm were approximately 1 nm, with no significant difference observed. In contrast, at 310 K, the blue shift in λ_max_ under Δλ = 15 nm (approximately 1.2–5.6 nm) was greater than that under Δλ = 60 nm (approximately 0.4 nm). This suggests that the increase in temperature weakens the binding between *α*-mangostin and NDH-2, resulting in enhanced hydrophobicity of the protein’s microenvironment. Furthermore, the data indicate that *α*-mangostin has a more pronounced effect on Tyr residues than on Trp residues.

### 2.5. Interaction Pattern Between α-Mangostin and NDH-2

**Figure 5:**
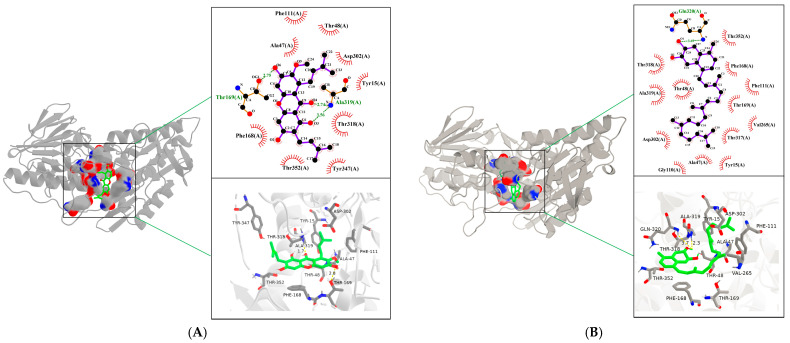
Molecular docking of α-mangostin and MK−4 respectively with NDH-2 (PDB: 5NA1). (**A**) 2D and 3D binding models of α-mangostin. (**B**) 2D and 3D binding models of MK. (Minor variations in interactions between 2D and 3D diagrams result from differences in computational algorithms).

Through molecular docking, we further investigated the binding site of *α*-mangostin with NDH-2. The docking results (Figure 5A) indicate that *α*-mangostin binds to the active center of NDH-2, with the docking site located the MK-binding pocket. Both the 2D and 3D docking diagrams clearly illustrate the types and strengths of interactions between *α*-mangostin and the amino acid residues in the binding region. Specifically, *α*-mangostin forms hydrogen bonds with two amino acids on NDH-2: a phenolic hydroxyl group forms a hydrogen bond with Thr169 (bond length: 2.75 Å), and the ketone carbonyl group forms a hydrogen bond with Ala319 (bond length: 2.74 Å). Additionally, hydrophobic interactions are observed with Tyr15, Asp302, Thr48, Thr318, Tyr347, Thr352, Phe168, Phe111, and Ala47. This hydrophobic cavity largely overlaps with the conserved cavity on the FAD si-side of NDH-2, which has been characterized as the quinone binding site [15]. In addition, the molecular docking of MK-4 and NDH-2 (Figure 5B) shows that *α*-mangostin occupies the same binding pocket as MK-4, with almost identical key interacting residues. The binding energy of *α*-mangostin from molecular docking is −6.56 kal/mol, while that of MK-4 is −7.21 kal/mol, and the difference in binding energy between the two is not significant. Therefore, these results further confirm that *α*-mangostin primarily competes with MK for binding at the active center of NDH-2.

### 2.6. Influence of Membrane Potential

**Figure 6:**
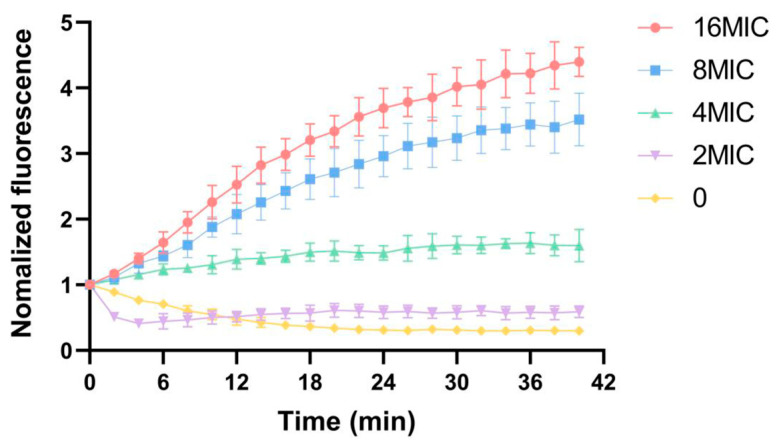
Depolarization of MRSA membrane potential by *α*-mangostin at concentrations of 2, 4, 8 and 16 × MIC. Experiments were performed as three biologically independent replicates, and data are presented as mean ± SD (*n* = 3).

DisC_3_(5) is a membrane potential-sensitive cyanine dye whose distribution between bacterial cells and the culture medium depends on the cytoplasmic membrane potential. When the dye enters cells, self-fluorescence quenching occurs due to increased concentration. Disruption of the cytoplasmic membrane structure leads to membrane potential dissipation, resulting in the release of the dye into the culture medium and a subsequent increase in fluorescence intensity. Specifically, the degree of membrane potential reduction is proportional to the increase in fluorescence intensity, and this change can be detected using a fluorescence spectrophotometer [20]. The addition of *α*-mangostin at different concentrations to bacterial suspensions induced an increase in DisC_3_(5) fluorescence intensity, indicating membrane potential collapse and the release of accumulated DisC_3_(5). This concentration-dependent relationship is shown in Figure 6—low concentrations of *α*-mangostin induced a gradual decline in membrane potential, while the fluorescence signal under high concentrations of *α*-mangostin exhibited a strong and immediate increase. These results demonstrate that *α*-mangostin significantly disrupts the membrane potential of *S. aureus*, inducing a depolarization effect.

## 3. Discussion

As a plant-derived flavonoid, *α*-mangostin exhibits broad pharmacological activities and has been demonstrated to possess potent inhibitory activity against Gram-positive bacteria [21]. The above results suggest that *α*-mangostin can compete with menaquinone to bind the active sites of NDH-2 of *S. aureus*, blocking the electron transfer into the quinone pool of the respiratory chain, affecting energy supply, and ultimately leading to bacterial autolysis under aerobic conditions. It is confirmed that the quinone pool in the respiratory chain is a major target of plant flavonoids against Gram-positive bacteria [11]. Therefore, this conclusion is further approved here.

In Gram-positive bacteria, menaquinone, as an electron receptor on the cell membrane, is the only quinone that performs electron transfer. After accepting the electrons from NDH-2, it is reduced to MKH_2_ which will transfer the electrons to the next enzyme in the respiratory chain, and this process also plays an important role for maintaining the energy for the proton gradient [13,22,23]. The analyses of fluorescence spectroscopy indicated that a significant quenching of the intrinsic fluorescence of NDH-2 occurred when incubated with *α*-mangostin. Remarkably, this quenching presented a concentration dependent pattern which is consistent with the Stern–Volmer equation, and accompanied by a blue shift in fluorescence peak positions and changes in fluorescence lifetime. This indicates there is direct binding between *α*-mangostin and NDH-2. By fitting the data, general parameters such as the binding constant, number of binding sites, enthalpy change, entropy change, and Gibbs free energy were obtained, indicating that the binding affinity decreases with the increase in temperature. This confirms that the quenching is static in nature, suggesting a spontaneous process of *α*-mangostin binding NDH-2. The binding constant (K_a_ < 10^4^ L·mol^−1^) indicates weak binding affinity, which seems to contradict the observed significant enzyme inhibitory effect. This is mainly because the binding constant reflects the overall binding capacity and cannot fully represent the specificity of target binding. Optimal enzyme inhibition depends on precise molecular recognition at the active site, rather than merely relying on the overall binding strength. Synchronous fluorescence spectroscopy shows that the effect of *α*-mangostin on the Tyr residues of NDH-2 is more significant than that on the tryptophan Trp residues. Molecular docking simulations reveal that Tyr residues are mostly distributed in the menaquinone-binding pocket. Therefore, it indicates that α-mangostin mainly affects the microenvironment of the MK pocket region.

Previous studies indicated that MK-4 or the MK extract from *S. aureus* can attenuate the antibacterial activities of plant flavonoids against *S. aureus*, and their antibacterial activities continuously decrease with the increase in the interfering concentrations of MK-4 and the MK extract, especially for *α*-mangostin [11]. This phenomenon occurs because the substrate both MK-4 and the MK extract can compete with *α*-mangostin for the enzyme active site, thereby reversing the inhibitory effect, which is exactly the hallmark characteristic of competitive inhibition. In the case of non-competitive inhibition, the addition of MK-4 or the MK extract should not produce such a competitive effect. Enzyme kinetic experiments show that *α*-mangostin exerts mixed-type inhibition on NDH-2, with the competitive mode being dominant in the mixed-type inhibition. Although the differences in inhibition constants derived from enzyme kinetic results are not highly significant, pure competitive inhibition requires that Kin→∞, which is extremely rare in reality. Moreover, multiple molecular docking demonstrates that the unique optimal binding site of α-mangostin is located at the menadione binding pocket. The conformation with the lowest binding free energy highly overlaps with the MK-binding site, and all key interacting residues are situated within the conserved quinone binding region. Molecular docking of MK-4 with NDH-2 also indicates that *α*-mangostin occupies the same binding pocket as MK-4, with nearly identical key interacting residues. It can be further inferred that *α*-mangostin exerts its inhibitory effect by competitively binding to the binding site of MK. These findings together suggest that *α*-mangostin can likely target the menaquinone-binding sites of NDH-2 to lead to the blocking of the binding of MK and NDH-2, rather than inhibiting the biosynthesis of MK. Namely, *α*-mangostin can compete with MK for the menaquinone-binding sites of NDH-2 of *S. aureus*.

It was reported that there are two independent binding sites in NDH-2, for the bindings of MK and NADH, respectively [15,24]. This research reveals that *α*-mangostin can bind to the MK-binding site of this protein. Simultaneously, under conditions where MK is saturated, the double-reciprocal plot of NADH as a substrate exhibits a nonlinear pattern in the presence of *α*-mangostin. Therefore, it can be inferred that *α*-mangostin specifically competes for the MK-binding site without interfering the binding of NADH. This is also supported by previous work which confirms that the engagement of drugs to MK-binding sites of NDH-2 does not directly affect the NADH binding process and that two binding sites are independent and functionally segregated [25].

Furthermore, this research showed that *α*-mangostin can cause the collapse of the membrane potential of *S. aureus*; this is in accordance with a previous report that *α*-mangostin can disrupt the cytoplasmic membrane and alter the proton motive force [10]. However, it had a low hemolysis rate on sheep red blood cells [26], with only 25.86% of red blood cells lysed at 32 μg/mL. *α*-Mangostin exhibits potent antibacterial activity, with the MICs ranging from 0.5 to 2 μg/mL and MBCs ranging from 4 to 64 μg/mL; the significant difference between MIC and MBC can be attributed to the collapse of membrane potential and subsequent disruption of the cell membrane. As a fungicide, α-mangostin can cause membrane damage after acting for a certain period of time, so its core target for killing *S. aureus* should be the quinone pool on the respiratory chain. These facts indicate that its damages on cytoplasmic membrane and proton motive force are more likely an apparent effect, rather than a core initial target [27,28]. Together with the consideration that menaquinone is the sole quinone in the quinone pool of *S. aureus*, it was deduced that *α*-mangostin mainly competes for the menaquinone-binding sites of NDH-2 to block the electron transfer at the quinone pool in the respiratory chain, hindering the energy supply of *S. aureus*, and also promoting its effect on membrane disruption, ultimately leading to a rapid bactericidal effect. Therefore, its antibacterial effect is the synergistic effect of targeting the quinone pool and damaging the cytoplasmic membrane since *α*-mangostin is a representative of plant flavonoids with very strong antibacterial activity against *S. aureus*, this finding once again proves that the quinone pool is a key target of plant flavonoids against Gram-positive bacteria, but also provides a new antibacterial mechanism of plant flavonoids.

## 4. Materials and Methods

### 4.1. Materials, Chemicals, and Reagents

*Escherichia coli* BL21(DE3) and the pET-28a(+) vector were purchased from Tsingke Biotechnology Co., Ltd. (Beijing, China). The strain of MRSA ATCC 33592 and *S.aureus* ATCC 25923 was obtained from the American Type Culture Collection (Manassas, VA, USA). *α*-Mangostin (purity > 98%, HPLC) was sourced from Push Bio-technology Co., Ltd. (Chengdu, China). Ni^2+^-NTA agarose resin was acquired from QIAGEN (Hilden, Germany). FAD (>98%, HPLC), NADH (>98%, HPLC), 3,3′-dipropylthiadicarbocyanine iodide DisC_3_(5) (>98%, HPLC) and menaquinone (MK) (>98%, HPLC) were all purchased from Sangon Biotech Co., Ltd. (Shanghai, China). Protein markers for gel analysis were obtained from Thermo Fisher Scientific (Waltham, MA, USA). Most chemical reagents used for NDH-2 expression, gel analysis, and Western blotting were supplied by Sangon Biotech Co., Ltd. (Shanghai, China), Sinopharm Chemical Reagent Co., Ltd. (Shanghai, China), or Xilong Scientific Co., Ltd. (Shantou, China). Mueller-Hinton Broth (MHB), purchased from Haibo Biotechnology Co., Ltd. (Qingdao, China), was used for antimicrobial susceptibility testing. *α*-Mangostin was dissolved in dimethyl sulfoxide (DMSO) and subsequently diluted to a final DMSO concentration of <2.5% (*v*/*v*) or lower.

### 4.2. Antimicrobial Susceptibility Assay

Following the standard procedures described by the Clinical and Laboratory Standards Institute (CLSI) [29], logarithmic phase bacterial cultures were diluted with Mueller-Hinton Broth (MHB) to a final concentration of approximately 1.0 × 10^6^ CFU/mL. The susceptibility of *S. aureus* ATCC 25923 and MRSA ATCC 33592 to *α*-mangostin was then determined using the microbroth dilution method in 96-well plates. After incubating the 96-well plates at 35 °C for 24 h in an incubator, 20 μL of 3-(4,5-dimethyl-2-thiazolyl)-2,5-diphenyltetrazolium bromide (MTT, 4.0 mg/mL) was added to each well. The minimum inhibitory concentration (MIC) was defined as the lowest concentration of the compound that completely inhibited bacterial growth in the wells, determined by the absence of color change when bacterial growth was fully observed in the blank control wells [30]. Based on the MIC results, wells with *α*-mangostin concentrations at different multiples of the MIC were selected. A 100 μL aliquot of bacterial suspension was aspirated from each selected well and spread onto solid medium plates, which were then incubated overnight. The minimum bactericidal concentration (MBC) was determined according to the number of bacterial colonies grown on the solid medium plates.

### 4.3. Expression and Purification of the Enzyme

Based on the obtained amino acid sequence WP 002463246.1 encoded by the *ndhC* gene, NDH-2 (NDH-2A) was expressed in *E. coli* BL21(DE3)/pET28a(+)-NDH-2. *E. coli* BL21(DE3)/pET28a(+)-NDH-2 was cultured on Luria-Bertani (LB) agar plates at 37 °C. NDH-2 single colony was picked and inoculated into fresh LB broth containing kanamycin (50 μg/mL), followed by incubation in a shaker at 37 °C and 180 rpm for 12 h. Subsequently, the culture was diluted 1:100 into fresh LB broth supplemented with kanamycin (50 μg/mL) and incubated at 37 °C until the optical density at 600 nm (OD_600_) reached 0.6–0.8. After cooling on ice for 15 min, isopropyl *β*-D-1-thiogalactopyranoside (IPTG) (Sangon Biotech (Shanghai) Co., Ltd., Shanghai, China) was added to a final concentration of 0.4 mM. The culture was then induced for expression in a shaker at 25 °C and 150 rpm for 10–12 h. After induction, bacterial cells were harvested by low-temperature centrifugation. Bacterial cells were resuspended in lysis buffer (50 mM Tris, 500 mM NaCl, 5% glycerol, 1 mM PMSF, pH 8.0) and lysed by ultrasonic disruptor. The supernatant was collected by centrifugation at 6000× *g* for 25 min, and loaded onto a column filled with Ni^2+^-NTA-agarose gel pre-equilibrated with buffer. Then, the column was washed with washing buffers containing different concentrations of imidazole. The purity of the purified protein was verified by SDS-PAGE, and the protein was identified by Western blotting. Using bovine serum albumin (BSA) as the standard, the protein concentration was determined with a BCA kit (Sangon Biotech (Shanghai) Co., Ltd., Shanghai, China).

### 4.4. Determination of NDH-2 Activity

The oxidation rate of NADH was determined in the presence of different concentrations of substrates. The 200 μL reaction mixture contained 20 μM FAD, 0.5 μg NDH-2, 5% glycerol, 150 mM NaCl, and 50 mM Tris-HCl buffer (pH 7.5). Under the incubation condition of 30 °C, enzyme kinetic parameters were measured by adding MK (0–60 μM) or NADH (0–450 μM). The reaction was initiated by the addition of NADH. The absorbance was monitored at 340 nm using a multifunctional microplate reader to track the reaction progress (NADH *ε* = 6.22 mM^−1^ cm^−1^), the experiment was repeated three times.

### 4.5. Inhibition of NDH-2 by α-Mangostin

The assay was performed using multifunctional microplate reader by measuring the absorbance at 340 nm. The reaction system was referenced to Section 4.4 of this research. To evaluate the inhibitory effect of *α*-mangostin on NDH-2, the enzyme was pre-incubated with various concentrations of *α*-mangostin in the reaction mixture for 2 min with 40 μM MK, and the reaction was initiated by the addition of 300 μM NADH. To analyze the inhibitory mechanism of *α*-mangostin on NDH-2, different concentrations of substrate MK (0–60 μM) were added to the reaction mixture. The enzymatic reaction rates were measured at varying substrate concentrations in the presence of *α*-mangostin (0, 1, 2 and 4 μg/mL). Lineweaver–Burk plots were generated to determine the inhibition type and inhibition constant (*K*_i_) of *α*-mangostin against NDH-2.

### 4.6. Fluorescence Spectroscopy Analysis of the Interaction Between α-Mangostin and NDH-2

Referring to the experimental method described by Zilong Luo et al. [31], fluorescence spectra of the interaction between *α*-mangostin and NDH-2 were measured using an FL970 fluorescence spectrophotometer (Taikang Instruments Co., Ltd., Shanghai, China). The slit width was set to 2.5 nm, the scan speed to 1200 nm/min, and the sampling interval to 0.2 nm.

The reaction system was prepared in PBS buffer (pH 7.4), containing 0.5 mg/mL NDH-2 and 20 μM FAD, with final concentrations of *α*-mangostin at 0, 1, 2, 4, 8, 10, 15 and 20 μg/mL, incubating the mixture at 298 K and 310 K for 5 min. The excitation wavelength was set to 280 nm, and fluorescence spectra were scanned in the range of 290–600 nm. Background fluorescence from the PBS buffer was subtracted, and the maximum emission wavelength and fluorescence intensity were recorded.

For the synchronous fluorescence spectra of the samples, measurements were performed at 298 K and 310 K. The excitation wavelength range was set to 250–500 nm, with wavelength differences (Δ*λ*) between emission and excitation wavelengths of 15 nm and 60 nm. The maximum emission wavelength and fluorescence intensity were recorded.

### 4.7. Molecular Docking

Molecular docking between *α*-mangostin, menaquinone−4 (MK-4) and NDH-2 was performed using AutoDock Tools 1.5 [32]. The crystal structure of NDH-2 (PDB ID: 5NA1) was downloaded from the RCSB PDB online database (https://www.rcsb.org/ (accessed on 1 September 2025)), and all crystal water molecules and metal ions were removed from the protein structure. FAD is retained as an indispensable structural cofactor. The three-dimensional structure of *α*-mangostin (PubChem SID: 433967754) was retrieved from the PubChem database (https://pubchem.ncbi.nlm.nih.gov/ (accessed on 1 September 2025)) and imported into Chem3D 22.2 for energy minimization. The active pocket of NDH-2 was predicted using the DrugRep tool [33]. The grid size was set to 32 Å × 20 Å × 32 Å, with the center coordinates of the docking box at X (14.8 Å), Y (14.2 Å), and Z (−24.7 Å), and a grid spacing of 0.375 Å. AutoDock Tools 1.5 was used to calculate the potential docking conformations of *α*-mangostin and MK-4 with NDH-2. We set the number of independent runs of the Lamarckian Genetic Algorithm (LGA) to 10, and finally obtained 10 independent docking results. The docking conformation with the lowest binding free energy was selected for binding mode analysis. The results were imported into PyMOL 2.6 and LigPlot+ 2.2.9 for further visualization and analysis, and to generate 3D and 2D docking interaction diagrams [34,35].

### 4.8. Measurement of Membrane Potential

The method described by Zhiqiang Wang et al. [36] was used with minor modifications. A single colony of MRSA ATCC 33592 was inoculated into MHB. Bacterial cells were harvested by centrifugation, washed with PBS buffer (pH 7.4), and resuspended in PBS buffer (plus 20 mM glucose). In a black 96-well plate, bacterial suspension and DisC_3_(5) (1 μM) were added, followed by incubation at 37 °C in the dark for 10 min. After monitoring until the baseline stabilized, different concentrations of *α*-mangostin (2, 4, 8 and 16 × MIC) was added. After incubation for 30 min, the fluorescence intensity was measured using a multifunctional microplate reader with an excitation wavelength of 622 nm and an emission wavelength of 670 nm.

## 5. Conclusions

In summary, this research demonstrates that *α*-mangostin mainly can interact with the amino acid residues at the menaquinone binding pocket of NDH-2 to block the electron transfer at the quinone pool in the respiratory chain of *S. aureus*. This will hinder the energy supply and promote its incidental effect on membrane disruption, ultimately leading to the death of *S. aureus*. Furthermore, this further supports the conclusion that the quinone pool is the key target of plant flavonoids inhibiting Gram-positive bacteria.

## Figures and Tables

**Table 1:** Parameters for the interaction between *α*-mangostin and NDH-2 obtained from fluorescence quenching at 298 K and 310 K ^a^.

Parameters	298 K	310 K
*K*_sv_ (×10^4^ L·mol^−1^)	5.28 ± 0.05	4.27 ± 0.05
*K*_q_ (×10^12^ L·mol^−1^·s^−1^)	5.28 ± 0.05	4.27 ± 0.04
R_a_	0.98	0.98
*K*_a_ (L·mol^−1^)	2.65 × 10^3^	646.7
*n*	0.70 ± 0.07	0.58 ± 0.06
R_b_	0.98	0.98
ΔH (kJ·mol^−1^)	−90.2	−90.2
ΔG (kJ·mol^−1^)	−19.5	−16.7
ΔS (kJ·mol^−1^·K^−1^)	−0.24	−0.24

^a^ *K*_sv_, quenching constant; *K*_q_, bimolecular quenching rate constant; *K*_a_, binding constant; *n*, number of binding sites; ΔH, enthalpy change; ΔG, Gibbs free energy change; ΔS, entropy change; R_a_ and R_b_ are the Pearson correlation coefficients for *K*_sv_, *K*_a_ and *n*.

## Data Availability

The data presented in this study are available on request from the corresponding author due to privacy.
